# The burden of pertussis in low- and middle-income countries since the inception of the Expanded Programme on Immunization (EPI) in 1974: a systematic review protocol

**DOI:** 10.1186/s13643-015-0053-z

**Published:** 2015-05-01

**Authors:** Rudzani Muloiwa, Benjamin M Kagina, Mark E Engel, Gregory D Hussey

**Affiliations:** Department of Paediatrics & Child Health, Red Cross War Memorial Children’s Hospital, University of Cape Town, Rondebosch, Cape Town 7700, South Africa; Vaccines for Africa Initiative, Division of Medical Microbiology & Institute of Infectious Disease and Molecular Medicine, University of Cape Town, Observatory, Cape Town, 7925 South Africa; Department of Medicine, University of Cape Town, Observatory, Cape Town, 7925 South Africa

**Keywords:** Pertussis, Whooping cough, Burden, Prevalence, Incidence, LMICs

## Abstract

**Background:**

Vaccine against pertussis has been in use for several decades. Despite the widespread use of pertussis vaccine, evidence shows resurgence of pertussis in high-income countries. Pertussis surveillance data is largely missing from low- and middle-income countries (LMICs). Without data on trends of pertussis, it is difficult to review and amend pertussis control policies in any country. We propose conducting a systematic review to evaluate the burden and trends of pertussis in LMICs since 1974.

**Methods/design:**

Common and medical subject heading (MeSH) terms for pertussis and LMICs will be used to search electronic databases for the relevant literature published between 1974 and December 2014. Only studies from LMICs that fulfils World Health Organisation (WHO) or CDC pertussis case definitions will be included. The studies must have a clear numerator and denominator in a well-defined population.

Risk of bias will be evaluated by assessing all qualifying full-text articles for quality and eligibility using a modified quality score assessment tool.

Standardised data extraction will be carried out after which descriptions of trends in the prevalence, incidence, as well as mortality rate and case fatality rate, will be done. Where sufficient data is available, the results will be stratified by age group, geography, location, vaccination and HIV status.

**Discussion:**

The systematic review proposed by this protocol seeks to address the knowledge gap in the epidemiology of pertussis in LMICs for the first time. It is anticipated that the background epidemiological trends of pertussis in LMICs that our study will provide could be used in the planning for the control of pertussis.

**Systematic review registration:**

PROSPERO CRD42015015159

## Background

Pertussis or whooping cough is a highly infectious respiratory illness caused by *Bordetella pertussis* or *Bordetella parapertussis*. Globally, the disease is notifiable in many countries. Worldwide, the World Health Organisation (WHO) estimates that 20 to 40 million annual cases of pertussis and 300,000 deaths occur due to the disease, of which 90% occur in low- and middle-income countries (LMICs) [1,2]. While good surveillance data support the re-emergence of pertussis in high-income countries, pertussis surveillance data is largely missing for LMICs. Therefore, trends in pertussis epidemiology in LMICs are unknown [3,4]. To strengthen the existing strategies of pertussis control in LMICs, it is critical to understand the background epidemiology of the disease in these settings. We are, therefore, conducting a systematic review to evaluate the burden of pertussis in LMICs.

The availability of an effective vaccine against *B. pertussis* since the 1940s led to a substantial reduction in the morbidity and mortality caused by pertussis [5]. In 1974, WHO included the whole cell vaccine (wP) in the Expanded Programme on Immunization (EPI). The wP is still widely used in many LMICs. However, in many high-income countries, the original wP has been replaced with various formulations of the acellular vaccine (aP), which are believed to be equally effective and with an improved safety profile compared to wP [6]. Some LMICs have also introduced aP to their national EPI schedules [7]. In the absence of complete pertussis epidemiology data in most of the LMICs, positive or negative impacts resulting from the introduction of aP will likely be missed. To improve any vaccination programme, monitoring and evaluation are crucial. A change in the epidemiology of a vaccine preventable disease is a key outcome in evaluation of a vaccination programme.

Epidemiological data from high-income countries show that, despite high vaccine coverage with aP, pertussis burden has increased in non-immunised or partially immunised infants [3,8]. Increasingly, in many settings, pertussis is being recognised as an important cause of morbidity in adolescents and adults, possibly due to waning immunity. Older individuals have less severe disease and fewer complications. However, older persons have substantial economic costs associated with unrecognised infection that may serve as an important source of infection for non-immune infants [9-11]. Evidence is emerging that aP vaccines may not have the same effectiveness or longevity of immunity as the wP vaccines; this may partly explain the observed re-emergence of pertussis in settings that have introduced the aP [3,8]. As many other LMICs consider the introduction of aP vaccines, understandably due to a superior safety profile, the epidemiological data of pertussis in these settings will be more and more critical.

The burden and trends of pertussis are a challenge in light of the difficulties in the diagnosis of the disease. The diagnosis of pertussis is largely made on the basis of the clinical picture. However, clinical presentation may be modified by age, previous immunisation or infection, antibiotic exposure and concurrent infection with other pathogens [5]. The presentation of pertussis is frequently atypical, especially in infants and adults [5]. Laboratory confirmation of cases by serology, culture or PCR is thus very important. One of the main reasons for lack of surveillance data in LMICs is absence of diagnostic facilities for confirmation of *B. pertussis*. This is compounded by a lack of standardised clinical case definition and lack of resources to support reporting of cases by health care workers [12]. Despite these challenges, there are some data on the epidemiology of the disease in a few LMICs that can be collated to foster better understanding and a snapshot of the pertussis disease in these settings. Our aim is to synthesise any available epidemiology data on pertussis in LMICs and present a snapshot of the pertussis burden in these settings. Our proposed systematic review will provide a baseline understanding of the disease burden and trends in LMICs.

### Rationale

The current epidemiology of pertussis and its changing pattern since the inception of the EPI in 1974 is poorly understood in LMICs. There are valid concerns that resurgence of pertussis may be happening in LMICs with possible future epidemics if no action is urgently taken. The high HIV prevalence in LMICs coupled with suboptimal vaccines uptake are risk-modifying factors that can fuel pertussis epidemics in these settings [13,14].

### Objectives

To conduct a systematic review and meta-analysis of studies assessing the prevalence of pertussis in LMICs since the inception of the Expanded Programme on Immunization (EPI) in 1974.

#### Primary objectives

1. To review available published literature on the incidence and/or prevalence of pertussis in LMICs since the inception of the EPI
2. To determine the trend in the incidence and/or prevalence of pertussis in LMICs from 1974 to 2014

#### Secondary objectives

1. To investigate the impact of HIV infection on the epidemiology of pertussis during the review period
2. To investigate the impact of vaccine coverage on the burden of pertussis in LMICs
3. To describe the mortality rate and case fatality rate ascribed to pertussis in LMICs

## Methods/design

### Eligibility criteria

#### Types of participants

The review will include studies involving both children and adults conducted in LMICs.

#### Case definition

Studies will be considered for inclusion only if they fulfil the pertussis case definitions recommended by WHO or/and CDC [15,16] as shown in the following:

WHO pertussis case definition

Clinical case definition

- ♦A case diagnosed as pertussis by a physician or
- ♦ A person with a cough lasting at least two weeks with at least one of the following symptoms:

- Paroxysms (that is, fits) of coughing
- Inspiratory whooping
- Post-tussive vomiting (that is, vomiting immediately after coughing) without other apparent cause

Criteria for laboratory confirmation

- ♦ Isolation of Bordetella pertussis or
- ♦ Detection of genomic sequences by means of the polymerase chain reaction (PCR) or
- ♦ Positive paired serology
- Case classification
- ♦ Clinically confirmed: A case that meets the clinical case definition but is not laboratory-confirmed
- ♦ Laboratory-confirmed: A case that meets the clinical case definition and is laboratory-confirmed

CDC clinical case definition

Clinical Case Definition

In the absence of a more likely diagnosis a cough illness lasting ≥2 weeks with one of the following symptoms:

- ♦ Paroxysms of coughing, OR
- ♦ Inspiratory ‘whoop,’ OR
- ♦ Post-tussive vomiting, OR
- ♦ Apnea (with or without cyanosis) (FOR INFANTS AGED < 1 YEAR ONLY)
- Laboratory Criteria for Diagnosis
- ♦ Isolation of *Bordetella pertussis* from clinical specimen
- ♦ Positive polymerase chain reaction (PCR) for *B. pertussis*
- Epidemiologic Linkage
- ♦ Contact with a laboratory-confirmed case of pertussis*.

Case Classification

Probable

Meets the clinical case definition, is not laboratory-confirmed, and is not epidemiologically linked to a laboratory-confirmed case, OR

FOR INFANTS AGED <1 YEAR ONLY

- ♦ Acute cough illness of any duration with at least one of the following signs or symptoms:

- Paroxysms of coughing, OR
- Inspiratory ‘whoop’, OR
- Posttussive vomiting, OR
- Apnea (with or without cyanosis)

AND

- ♦ Polymerase chain reaction (PCR) positive for pertussis, OR

FOR INFANTS AGED <1 YEAR ONLY:

- ♦ Acute cough illness of any duration with at least one of the following signs or symptoms:

- Paroxysms of coughing, OR
- Inspiratory ‘whoop’, OR
- Posttussive vomiting, OR
- Apnea (with or without cyanosis)

AND

- ♦ Contact with a laboratory-confirmed case of pertussis

Confirmed

- ♦ Acute cough illness of any duration with isolation of *B. pertussis* from a clinical specimen, OR
- ♦ Meets the clinical case definition AND is polymerase chain reaction (PCR) positive for pertussis, OR
- ♦ Meets the clinical case definition AND had contact with a laboratory-confirmed case of pertussis

*Note: An illness meeting the clinical case definition should be classified as ‘probable’ rather than ‘confirmed’ if it occurs in a patient who has contact with an infant aged <1 year who is polymerase chain reaction (PCR) positive for pertussis and has ≥1 sign or symptom and cough duration of <14 days.

#### Inclusion criteria

- Both adults and children will be included
- Clear diagnostic criteria
- All clinical and population studies with clear denominator

#### Exclusion criteria

- Published before 1974 and after 2014
- Unclear denominator derivation
- Unclear diagnostic criteria
- Case reports
- Not conducted in LMICs

### Outcomes

#### Primary outcomes

##### *Prevalence or incidence of pertussis*

Incidence will be defined as events of pertussis occurring over the total period participants are at risk. Prevalence will be defined as proportions of all participants suspected of pertussis with confirmed laboratory diagnosis or proportions of participants fulfilling a clinical case definition.

#### Secondary outcomes

Secondary outcomes will include vaccination status, mortality and case fatality rates associated with pertussis as well as the HIV prevalence in the study settings of the included studies.

Vaccination status will be classified as follows: unknown, where no immunisation information is provided; unvaccinated, where data indicates that no single vaccine dose has been received; partially vaccinated, where data indicates an incomplete primary schedule (less than three doses in the first year of life); or fully immunised (completed primary schedule with or without a booster dose).

Mortality will be defined as the proportion of deaths attributable to pertussis in a study population as a proportion of all deaths while case fatality will be defined as mortality among confirmed or probable cases of pertussis.

#### Type of studies

Cross sectional, cohort and surveillance studies will be included. Both prospective and retrospective studies will be included. Inpatient, outpatient and population-based studies will be included provided a denominator is available with explanation as to how it was derived.

The studies must have been conducted from 1974 until the end of 2014. The starting period was chosen as it is the year in which WHO formally initiated the EPI.

### Search strategy methods for the identification of studies

A comprehensive and sensitive search strategy has been developed to identify relevant studies available from 1 January 1974 through 31 December 2014, regardless of language or publication status (published, unpublished, in press or in progress).

Several electronic databases will be searched for all the relevant publications. Specifically the following databases will be searched:

1. MEDLINE via PubMed
2. Scopus
3. Africa-Wide
4. PDQ-Evidence
5. WHOLIS
6. CINAHL
7. CENTRAL

We will use both text words and medical subject heading (MeSH) terms; for example, whooping cough OR pertussis, epidemiology, incidence and prevalence. These terms will be combined with other relevant terms including an African filter [17]. The literature search strategy will be adapted for each database. Table 1 shows the search strategy that we have developed for use in PubMed.

**Table 1:** **Search strategy and output from PubMed**

**Query number**	**Search term**
#1	Pertussis (MeSH) OR whooping cough(MeSH)
#2	Bordetella pertussis OR B. pertussis OR Bordetella parapertussis OR B. parapertussis
#3	#1 OR #2
#4	Burden OR epidemiology OR incidence OR prevalence OR case*
#5	#3 AND #4
#6	(Angola OR Republic of Angola OR Albania OR Republic of Albania OR Algeria OR The People’s Democratic Republic of Algeria OR American Samoa OR Argentina OR Azerbaijan OR Belarus OR Belize OR Bosnia and Herzegovina OR Bosnia-Herzegovina OR Bosnia OR Botswana OR Brazil OR Federative Republic of Brazil OR Bulgaria OR China OR People’s Republic of China OR Colombia OR Costa Rica OR Fiji OR Gabon OR Gabonese Republic OR Grenada OR Hungary OR Islamic Republic of Iran OR Persia OR Iran OR Iraq OR Jamaica OR Jordan OR Hashemite Kingdom of Jordan OR Kazakhstan OR Lebanon OR Lebanese Republic OR Libya OR State of Libya OR Macedonia OR Republic of Macedonia OR Malaysia OR Maldives OR Republic of the Maldives OR Maldive Islands OR Marshall Islands OR Republic of the Marshall Islands OR Palau OR Republic of Palau OR Panama OR Republic of Panama OR Peru OR Romania OR Serbia, OR the Republic of Serbia OR Seychelles OR the Republic of Seychelles OR South Africa OR Saint Lucia OR Saint Vincent and the Grenadines OR Suriname OR Thailand OR Kingdom of Thailand OR Tonga OR Kingdom of Tonga OR Tunisia OR Turkey OR Turkmenistan OR Turkmenia OR Cuba OR Dominica OR Commonwealth of Dominica OR The Dominican Republic OR Ecuador OR Mauritius OR Mexico OR United Mexican States OR Montenegro OR Namibia OR Tuvalu OR Ellice Islands OR Venezuela OR the Bolivarian Republic of Venezuela)
#7	(Armenia OR armenia OR Bhutan OR Kingdom of Bhutan OR Bolivia OR Plurinational State of Bolivia OR Cameroon OR Republic of Cameroon OR Republic of Cameroun OR Cape Verde OR Republic of Cape Verde OR Cote D’ivoire OR Ivory Coast OR Republic of Cote D’ivoire OR Djibouti OR Republic of Djibouti OR Arab Republic of Egypt OR Egypt OR El Salvador OR Georgia OR Ghana OR Republic of Ghana OR Guatemala OR Republic of Guatemala OR Guyana OR Co-operative Republic of Guyana OR Honduras OR Republic of Honduras OR Spanish Honduras OR Republic of Indonesia OR Indonesia OR India OR Republic of India OR Kiribati OR Republic of Kiribati OR Kosovo OR Kosovo and Metohija OR Laos OR Lao Lao People’s Democratic Republic OR Lesotho OR Kingdom of Lesotho OR Mauritania OR Islamic Republic of Mauritania OR Micronesia, Fed. Sts. OR Federated States of Micronesia OR FSM OR Moldova OR Republic of Moldova OR Mongolia OR Morocco OR Kingdom of Morocco OR Nicaragua OR Republic of Nicaragua OR Nigeria OR Federal Republic of Nigeria OR Pakistan OR Islamic Republic of Pakistan OR Papua New Guinea OR Independent State of Papua New Guinea OR Paraguay OR Republic of Paraguay OR Philippines OR Republic of the Philippines OR Samoa OR Independent State of Samoa OR Sao Tome and Principe OR Democratic Republic of Sao Tome and Principe OR Senegal OR Republic of Senegal OR Solomon Islands OR Sri Lanka OR Democratic Socialist Republic of Sri Lanka OR Sudan OR Republic of the Sudan OR North Sudan OR Swaziland OR Kingdom of Swaziland OR Ngwane OR Yuwatini OR Syrian Arab Republic OR Syria OR East Timor OR Timor-leste OR Democratic Republic of Timor-leste OR Ukraine OR Uzbekistan OR Republic of Uzbekistan OR Vanuatu OR Republic of Vanuatu OR Vietnam OR the Socialist Republic of Vietnam OR West bank and Gaza OR Yemen OR Yemeni Republic OR Zambia OR Republic of Zambia)
#8	(Afghanistan OR Islamic Republic of Afghanistan OR Bangladesh OR People’s Republic of Bangladesh OR Benin OR Dahomey OR Republic of Benin OR Burkina Faso OR Burkina OR Republic of Upper Volta OR Burundi OR Republic of Burundi OR Cambodia OR Kingdom of Cambodia OR Central African Republic OR Chad OR Republic of Chad OR Comoros OR Union of the Comoros OR Democratic Republic of the Congo OR DR Congo OR Congo-Kinshasa OR DRC OR Zaire OR Eritrea OR State of Eritrea OR Ethiopia OR Federal Democratic Republic of Ethiopia OR The Gambia OR Republic of the Gambia OR Guinea OR Republic of Guinea OR Guinea-Conakry OR Guinea-Bissau OR Republic of Guinea-Bissau OR Haiti OR Republic of Haiti OR Kenya OR Republic of Kenya OR North Korea OR Democratic People’s Republic of Korea OR Kyrgyz Republic OR Kyrgyzstan OR Liberia OR Republic of Liberia OR Madagascar OR Republic of Madagascar OR Malawi OR Republic of Malawi OR The Warm Heart of Africa OR Mali OR Republic of Mali OR Mozambique OR Republic of Mozambique OR Myanmar OR Burma OR Republic of the Union of Myanmar OR Nepal OR Democratic Republic of Nepal OR Niger OR Republic of Niger OR Rwanda OR Republic of Rwanda OR Sierra Leone OR Republic of Sierra Leone OR Somalia OR Federal Republic of Somalia OR South Sudan OR Republic of South Sudan OR Tajikistan OR Republic of Tajikistan OR Tanzania OR United Republic of Tanzania OR Republic of Tanganyika and Zanzibar OR Togo OR Togolese Republic OR Uganda OR Republic of Uganda OR Zimbabwe OR Republic of Zimbabwe OR Rhodesia)
#9	#6 OR #7 OR #8
#10	#5 AND #9

### Selection of eligible studies

The primary author (RM) will screen the search outputs using titles and abstracts. In addition, publication date, study setting, study design, methods and patient population as well as study outcomes will be evaluated. The first and the second authors will then independently go through the full text of all potentially eligible studies to assess whether they meet the inclusion criteria. Discrepancies in the list of included studies between the two authors will be resolved through discussion and consensus, with the assistance of the other two authors.

### Data collection process

Data will be extracted from text, tables and figures and recorded on a standardised form. Study investigators will be contacted in cases of unclear data or eligibility criteria. Among others, the following data will be extracted from studies meeting our eligibility criteria:

- Study characteristics: period, design, objectives and inclusion criteria
- Study population: country, setting, type of facility and denominator
- Diagnostic methods: laboratory methods and clinical case definitions
- Prevalence or incidence of pertussis: confirmed cases and cases making clinical definition
- Pertussis patient characteristics: age and gender, HIV status and pertussis vaccination status
- Pertussis deaths: mortality and case fatality rate

### Risk of bias in individual studies

We will adapt the tool developed by Wasserman *et al*. to assess the risk of bias as well as the quality of the included studies (Table 2) [18]. This assessment criterion is comprehensive and sufficient for the different types of study designs that will be selected for inclusions. In brief, specific variables will be examined to assess internal and external validity of the selected studies. The tool takes into account the issues and principles discussed by Hoy *et al.* pertaining to internal and external validity of prevalence studies [19].

**Table 2:** **Tool for assessment of quality and bias**

**Item**	**Score**
1. Study design (selection score)	
a. Prospective clinical studies (any type)	
♦ Consecutive enrolment	2
♦ Unspecified/random enrolment	1
b. Autopsy studies	
♦ Consecutive enrolment	2
♦ Unspecified/random enrolment	1
c. Retrospective reviews (including subgroup analysis)	1
d. Review/editorial	0
e. Case report	0
2. Study objectives (selection score)	
a. Clear inclusion criteria	
♦ Aim related to pertussis prevalence or outcomes	3
♦ Related to OIs/general morbidity/respiratory disease but not specific to pertussis	2
♦ Unrelated to clinical pertussis prevalence	1
b. Unclear inclusion criteria	1
3. Diagnosis* (quality of outcome ascertainment)	
a. Laboratory-confirmed (any specified method/specimen)	2
b. Clinical case definition (consistent with WHO or CDC guidelines)	1
c. Not specified	0
4. Denominator	
a. Raw data denominator	2
b. Calculated denominator	1
c. No/unclear denominator/investigated < % of cohort for pertussis/exclusion group	0
5. Numerator (Pertussis numbers)	
a. Raw data numerator	2
b. Calculated numerator	1
c. No/unclear numerator (clinical pertussis not mentioned/tested)	0
6. Assessment of bias (low, high or unclear risk)	
a. Attrition bias	
♦ Amount, nature or handling of incomplete outcome data	
b. Selection bias	
♦ Representativeness of the cases/cohort (clear reasons for and rates of non-inclusion)	

*Do not include data from clinical episodes of pertussis if no case definition provided (even if study otherwise qualifies). CDC, Centers for Disease Control; OI, opportunistic infection; WHO, World Health Organisation.

### Data synthesis

Data will be summarised using incidences and/or prevalence.

The heterogeneity of the included studies will be evaluated using the *χ*^2^-based Q statistic (significant for *P* < 0.1) and the *I*^2^ statistic [20]. Where sufficient homogeneity (*I*^2^ statistic < 50%) between studies exists, the data will be pooled in a meta-analysis using a Mantel-Haenszel random effects model.

Subgroup analysis will be conducted based on the income level of the country as defined by criteria set down by the World Bank using the World Bank Atlas method (low, lower and upper middle-income countries) [21,22]. Other variables that will be considered for subgroup analysis are age group, HIV status (if indicated), HIV prevalence in the study country at the time of the study, diagnostic criteria, period in which the study took place and vaccination coverage as well as the location of the study (hospital versus population based).

Proportions as percentages will be used to depict vaccination status, case fatality rate and HIV prevalence while deaths per 100,000 population will be used to represent mortality rate.

The overall effect of study sample size on the study outcomes will be assessed through sensitivity testing.

STATA software version 13 (STATA Corporation, College Station, TX, USA) will be used to perform all the statistical calculations.

The study will utilise the guidelines for reporting systematic reviews as set down by the revised 2009 PRISMA statement. Findings in our systematic review will be presented in several ways. Flow diagrams will be used to summarise the study selection process including a list of excluded studies with reasons for exclusion. Tables for both individual studies as well as for summaries of all included studies will be used to present quantitative data. Qualitative data such as assessment of quality of studies and strength of evidence will be described in the text.

## Discussion

Although high pertussis vaccination coverage has been achieved in many countries, data from developed countries show that the pertussis burden, particularly among adolescents and adults, has increased during the last decade, and this presents an increased risk to infants prior to complete vaccination [10]. In contrast, pertussis burden in LMICs is less understood. For every country, sound understanding of the trends in the burden of pertussis is required to assess the impact of current pertussis control policies as well as decide on future policy direction. We anticipate that results from our study will provide a snapshot of the background epidemiological trends of pertussis in LMICs. This information could be used in disease control planning.

It is presumed that most of the burden of pertussis occurs in LMICs [2]. However, there is no systematised evidence of the burden of the disease as well as the impact that vaccination against pertussis has had since the inception of EPI in 1974. Of concern, high-income countries that routinely report the least burden of vaccine preventable diseases have lately been reporting pertussis resurgence resulting in the review of disease control policies [8,3]. It is likely that LMICs will also need a review of the existing pertussis control programmes. The systematic review and its envisaged meta-analysis that this protocol proposes seek to address the knowledge gap in the epidemiology of pertussis in LMIC for the first time and in a systematic approach.

### Protocol registration

The protocol has been published in the PROSPERO International Prospective Register of systematic reviews (http://www.crd.york.ac.uk/PROSPERO), registration number CRD42015015159.

## Abbreviations

aP: acellular vaccine
EPI: Expanded Programme on Immunization
HIV: human immunodeficiency virus
LMICs: low- and middle-income countries
MeSH: medical subject heading
wP: whole cell vaccine
